# Interventions to Promote Positive Affect and Physical Activity in Children, Adolescents and Young Adults—A Systematic Review

**DOI:** 10.3390/sports8020026

**Published:** 2020-02-20

**Authors:** Leon Klos, Katharina Feil, Tanja Eberhardt, Darko Jekauc

**Affiliations:** Institute of Sports and Sports Science, Karlsruhe Institute of Technology, 76131 Karlsruhe, Germany; leonklos@gmx.de (L.K.); katharina.feil@gmx.net (K.F.); tanja.eberhardt@student.kit.edu (T.E.)

**Keywords:** physical activity, enjoyment, intervention, children, adolescents, affect, emotion, self-determination theory

## Abstract

Interventions to promote physical activity (PA) in children, adolescents and young adults based on social-cognitive theories often fail to increase PA. In recent years, affect-based approaches have gained interest, but the current state of research is not sufficiently reported. Therefore, a systematic review about the influence of interventions to promote positive affect and PA enjoyment and PA in children, adolescents and young adults was conducted. Literature searches were carried out including studies published between September 2009 and April 2019. Intervention studies targeting healthy children, adolescents or young adults and measuring enjoyment and PA were included. Thirteen studies met the inclusion criteria, including five group-based PA interventions, three multi-component school interventions, two internet-based interventions and three exergaming interventions. Most studies use multiple components in their intervention. Group-based PA programs incorporating task-oriented teaching styles and opportunities for voluntary PA are most consistently associated with positive findings. This review shows moderate evidence of interventions for children, adolescents and young adults being effective in increasing enjoyment and PA. Besides physical education and comprehensive school interventions, heterogenous intervention designs limit the comparability of studies. Future research should focus on theory-based, multi-component interventions with mediator analyses.

## 1. Introduction

Although health benefits of physical activity (PA) are widely known [1], only a minority of people meet the international PA guidelines of the World Health Organization [2,3]. This deficit is already observed in childhood. In Germany, 74% of children and adolescents aged 3 to 17 years do not fulfill the international recommendations of 60 minutes of PA every day [4]. These children and adolescents have an increased vulnerability for obesity, coronary heart disease and depression [5]. Additionally, physical activity is able to strengthen psychological and social resources such as self-esteem, higher confidence and social skills [6].

Over the years, different models have been developed to design interventions targeting PA behavior. Whilst the social-ecological model [7] not only aims at the development of personal skills but also at the socio-environmental context of an individual, the effectiveness of community actions remains unclear due to the difficulty of implementation in intervention studies [8,9]. Focusing on individual behavior, theories such as the social cognitive theory and the theory of planned behavior are based on the assumptions that the intention to perform a behavior predicts future behavior and that these are again influenced by constructs such as self-efficacy, attitudes and subjective norms. Whilst these theories are more applicable to PA interventions, those interventions often fail to increase PA, and only small amounts of variance of PA behavior of children and adolescents are explained by these theories [10,11]. Therefore, the use of such theories is not sufficient for designing effective interventions. In contrast to these rational approaches, a new, affect-based paradigm has emerged. According to the affect, heuristic positive and negative affective feelings influence judgement and decision making without the involvement of cognition [12]. It was shown that positive feelings lead to higher participation in sport than negative feelings [13]. In other words, people tend to repeat their behavior if they experience joy and fun [14]. Consequently, a focus on positive affective experiences of PA promises to be a new approach in health promotion.

The usage of “emotion” and “affect” is different depending on the spoken language. In English literature, “affect” conducts itself as an umbrella term for terms like feeling, emotion and mood. In psychological literature, categorical and dimensional models of emotions have been differentiated. Categorical models propose a discrete number of emotional categories. For example, Ekman [15] determined seven basic emotions (fear, contempt, sadness, happiness, surprise, anger and disgust) based on human fundamental life-tasks. Dimensional models conceptualize human emotions on two or three dimensions. For example, Russell [16] proposed the circumplex model, in which emotions are located on a continuum of valence (pleasure–displeasure) and arousal (sleepy–activated). These feelings proceed from the core affect, which is defined as “a neurophysiological state that is consciously accessible as a simple, nonreflective feeling (…)” [16]. 

The affective-reflective theory of physical inactivity by Brand and Ekkekakis [17] is based on dual process theories. These interpret behavior as a result of two mental processes, one fast and automatic process, and one slow and reflective process [18]. The affective-reflective theory states that the combination of affective valuation (fast and automatic) and the proposition to be physically active (slow and reflective) are related to action plans and PA behavior [17].

The self-determination theory (SDT) represents another approach to understand exercise behavior and demonstrates the importance of intrinsic motivation for PA [19]. Intrinsic motivation is linked to the three basic psychological needs: competence, relatedness and autonomy, which have been found to correlate with affective responses like pleasure and enjoyment in a PA context [20,21,22]. Perceived physical competence can influence enjoyment in physical education (PE) [20,23]. Further, autonomy [21] and relatedness [24] can predict PA enjoyment in adolescents. Leisterer and Jekauc [25] identified emotional triggers during PE lessons, three of which match the basic psychological needs. In another experimental study, they tested whether the manipulation of competence and social integration influenced affective judgements in the context of PE. They concluded that intrinsic motivation and positive emotions can be traced back to similar needs and triggers [26]. 

In explorative studies, different theories are used to describe the relation between affective response and PA. Based on the theory of planned behavior (TPB), Kwan and Bryan [27] found that persons with positive affective responses reported more favorable attitudes, higher self-efficacy and intentions to exercise. Other frameworks, like the social ecological model, seem attractive for intervention studies focusing on PA behavior [28]. Moreover, new theories like the affect and health behavior framework highlight new aspects (e.g., affect processing and affectively charged motivation) to be taken into consideration for interventions to promote positive affect [29].

Current research supports a relation between affective responses and PA. In a review of Rhodes and Kates [30], positive changes in affect were found during exercise. Additionally, this affective experience was linked to future PA. Besides this direct pathway, mediator analysis would be useful to detect influencing factors on affect and the relation to PA. It is still unclear how affective experiences can be manipulated to foster behavior change. Besides self-efficacy, affective judgement seems to be a reliable correlate of PA. For children and adolescents, a meta-analysis with 56 non-experimental studies yielded a small effect size (*r* = 0.26) [31]. A larger effect was found for adults (*r* = 0.42) [32]. Because of small sample sizes and heterogeneity of studies, a clear implication for future intervention studies is missing. Some interventions carried out in a school setting are able to increase PA enjoyment and show small-to-moderate effect-sizes [33]. PA enjoyment improved significantly in five out of the ten studies included in the meta-analysis of Burns et al. [33]. Although some interventions can change PA enjoyment of children and adolescents, the evidence of these interventions affecting enjoyment and PA behavior is limited to very few interventions using a wide range of different approaches and components [31].

Past reviews have mainly focused on non-experimental studies or did not analyze PA as a separate outcome variable. The review by Nasuti and Rhodes [31] on affective judgement and PA in children and adolescents only included literature published between 1990 and 2011. Additionally, no comprehensive review has focused on young adults as a specific target group. Therefore, a review is necessary to assess the progress and development of the field in order to bring the evidence together and establish general recommendations for more targeted research. With that, progress can be made to present evidence-based approaches to policy makers and professionals [34].

Therefore, this systematic review aims to (1) asses the effectiveness of interventions that promote positive affect and PA in children, adolescents and young adults, and (2) propose guidelines for future studies based on the evidence gathered in the first step. We hypothesized that interventions to promote positive affect and PA will influence PA enjoyment. Further, we assumed that interventions to promote positive affect and PA will influence PA behavior.

## 2. Materials and Methods

This systematic review was conducted according to the Preferred Reporting Items for Systematic Reviews and Meta-Analyses (PRISMA) guidelines [35].

### 2.1. Eligibility Criteria

Studies meeting the following inclusion criteria were included in this review: (1) a measurement of PA with PA defined as “any bodily movement produced by skeletal muscles that results in energy expenditure” [36], (2) a measurement of affective judgement towards PA, (3) intervention focusing on increasing PA and positive emotions towards PA and (4) participants who were children, adolescents under the age of 18 years or college students. Only published papers in peer-reviewed journals written in English were considered. Exclusion criteria were study populations defined by their physical disease, like cancer or diabetes. Additionally, study populations with mental illness, like depression, schizophrenia or dementia, were excluded.

### 2.2. Search

The databases Web of Science and Pubmed were searched for papers published between September 2009 and April 2019. A preliminary analysis in other psychological and sport scientific databases revealed a high number of duplicates, which is why the chosen databases were evaluated as the most comprehensive ones, including many different journal domains. The last search was conducted on 1 April 2019. Our search term consisted of four types of related terms:

- Physical activity-related terms: “physical activity” OR exercise OR sport*
- Emotion-related terms: emotion* OR affect* OR feel* OR enjoy* OR fun OR liking OR pleasure OR “physical activity enjoyment scale” OR PACES
- Study design: intervention OR “controlled trial”
- Mental disease-related terms: NOT (depress* OR schizophren* OR dementia* OR alzheimer* OR addict*)

Terms marked with an asterisk (*) include all words starting with the preceding letters. Of the first three term types, at least one term has to be met, and mental disease-related terms must not occur. Furthermore, we screened reference-lists and citations of eligible studies to identify additional relevant studies. 

### 2.3. Study Selection

The search was executed stepwise by three independent reviewers. The data was managed with Citavi 6.3 (Swiss Academic Software GmbH, Wädenswil, Switzerland). In the first selection step, a screening of titles was carried out. In the next step, abstracts were inspected for eligibility. Abstracts meeting the criteria were further examined by reading the full text articles. Full texts were also read for studies that had abstracts providing insufficient information about eligibility. Potential studies for inclusion in the review were scanned by at least two reviewers. Disagreements regarding inclusion were solved by discussion. Also, a third independent opinion of a reviewer was considered. Consensus was achieved in 100% of the cases. 

### 2.4. Data Extraction

We extracted the data from full text articles and related publications, when it was necessary. The data was filled into a five-item extraction form which contains source (authors, year of publication, country of origin), study design (theory, measurement points, statistics), sample (setting, sample size, mean age), intervention design (length of intervention, treatment, duration, frequency), outcome (measurements of affective judgement and PA) and results. Additional measurements and results (such as self-efficacy) that are not relevant to the research question are not shown.

### 2.5. Quality Assessment

Studies were assessed independently by two reviewers via the Effective Public Health Practice Project (EPHPP) assessment tool (McMaster University, Hamilton, Ontario, Canada) to grade the study quality. The EPHPP assessment tool is a standardized method used for quantitative studies in public health-related research. This tool incorporates selection bias across participants, study design, confounders, blinding of researchers and participants, data collection methods and withdrawals and drop-outs, into a global quality rating which differentiates between weak, moderate and strong. Strong quality was considered when none of the items were graded as weak, moderate quality was considered with one weak rating and weak quality was considered with two or more weak ratings [37]. Discrepancies in the evaluation of items were successfully resolved by discussion in all of the cases.

## 3. Results

**Figure 1:**
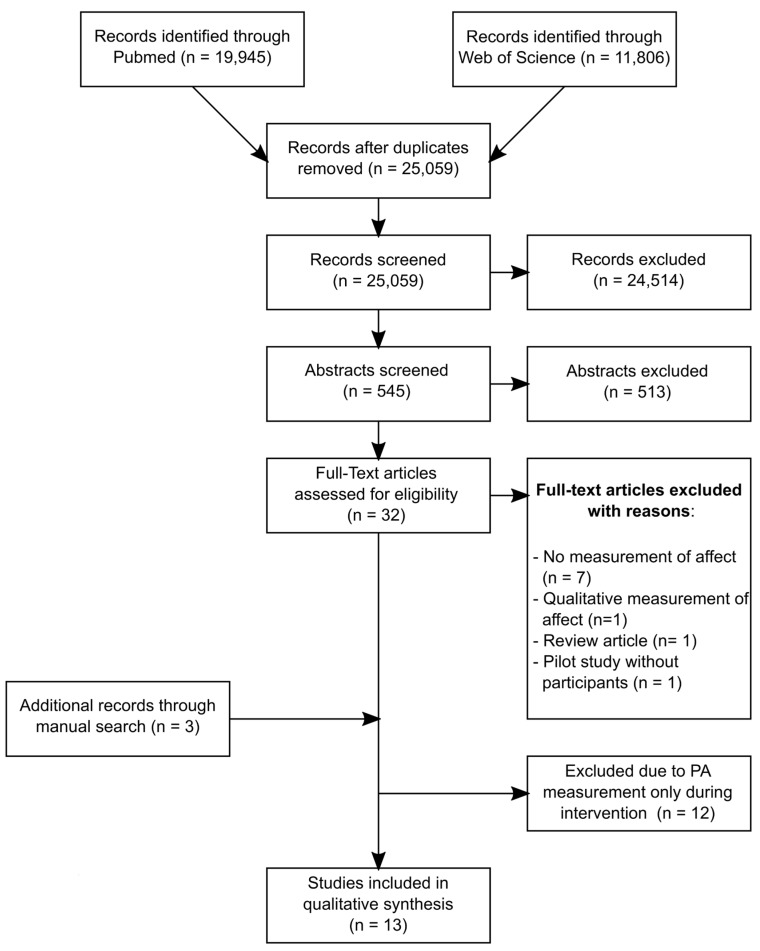
Results of the literature research, 2009–2019.

After removing duplicates, a total of 25,059 studies were identified. 545 studies remained after screening titles. Abstract screening yielded 32 full text articles assessed for eligibility. During full text reading, ten studies were excluded for not meeting the inclusion criteria. A further twelve studies did not sufficiently measure PA during the intervention time and were therefore eliminated. Three additional studies were added through manual research by checking references from included studies (see Figure 1). In total, 13 studies were included.

### 3.1. Study Characteristics

Six studies were conducted in North America [38,39,40,41,42,43], five studies in Europe [44,45,46,47,48], one in Singapore [49] and one in New Zealand [50]. Sample sizes ranged from 15 [39] to 1519 [43], and the duration of interventions ranged from three weeks [46] to three years [45]. Further study characteristics are presented in Table 1.

### 3.2. Study Quality

The analysis of study quality revealed one study classified as strong [43], nine studies as moderate [39,40,41,42,44,45,46,48,50] and three studies as weak [38,47,49]. Due to missing documentation of confounders prior to the intervention or insufficient control of confounders, most of the studies resulted in a weak rating. This leads to a downgrade in the global rating of these studies. All measurement instruments were rated as reliable and valid tools. Three studies did not report withdrawals nor dropouts [38,47,48].

### 3.3. Content of Interventions

Overall, eight studies were identified as having achieved significant positive changes in at least one outcome variable. The interventions of six studies were only able to significantly change enjoyment [40,43,44,45,46,49], while five studies increased PA solely [38,44,45,46,49]. Four interventions reported changes in both variables [44,45,46,48].

Most interventions employ multiple strategies targeting enjoyment and PA. These include, in descending order of commonness, group-based PA [40,41,42,44,48,49], implementing different teaching styles focusing on task-orientation [44,45,47,48], voluntary, supervised PA (e.g., during recess) [43,45,47,49], free access to exercise and sports facilities and equipment [45,47,49], internet-delivered motivational messages and information [43,46,49], video- and audio-based exergames [38,39,50] and group discussions and storytelling of PA-related topics [40,42]. Components only used in single studies are face-to-face motivational interviewing sessions [43], sharing progress on social media [49] and tracking progress via a pedometer [46]. A complete description of the studies can be found in Table 2.

#### 3.3.1. Group-Based PA Programs

Five studies centered their intervention around group-based PA programs for children [40,44,48], adolescents [42] and university students [41]. Two interventions substituted PE lessons for structured PA programs emphasized on flexible teaching strategies enabling enjoyable and inclusive activities and active reflection [44,48]. Both report higher enjoyment (η² = 0.96, *p* < 0.001) [48] as well as PA (*d* = 0.18, *p* = 0.033; η² = 0.09, *p* = 0.033) in comparison to regular PE [44,48]. The very high effect on enjoyment in the study of Invernizzi et al. [48] should be interpreted with caution. Two studies targeting girls only combined their PA program with group discussions [40,42]. Huberty et al. [40] linked storytelling and discussion to the following PE lesson, archiving a significant increase in enjoyment at follow-up. The other study missed such link and only used dance aerobic as group-based PA, not increasing enjoyment and decreasing PA significantly [42]. A lifetime physical fitness course for university students, which did not specify affect-based components, was not able to change enjoyment nor PA [41].

#### 3.3.2. Multi-Component School Programs

Three studies used comprehensive school interventions for older children, one of which focused solely on girls [43]. All interventions created new opportunities for PA in the form of organized PA during recess or after school [43,45,47], providing sport equipment [45,47] and access to fitness halls [47]. In two studies, PE teachers were trained to change their teaching style according to different theories in order to increase their students’ motivation and enjoyment for PA [45,47] and one additionally provided PA information materials [45]. In contrast, Robbins et al. [43] provided two motivational interviewing sessions with a health professional, and an internet-based session with individually tailored messages to encourage PA. One study conducted a mediator analysis showing a positive intervention effect on enjoyment (*B* = 0.07, *p* = 0.047) which improved moderate-vigorous physical activity (MVPA) (*B* = 24.48, *p* < 0.001) even though the intervention failed to change MVPA overall [43]. The intervention of Murillo Pardo et al. [45] increased both enjoyment (*β* = 0.11, *p* < 0.001) and MVPA (*β* = 8.51, *p* < 0.001), with enjoyment as a predictor of MVPA approaching significance (*β* = 2.22, *p* = 0.080) [45]. Gråstén and Yli-Piipari [47] found no significant intervention and no relation between enjoyment and MVPA.

#### 3.3.3. Internet-Based Programs

Two studies used internet-based interventions to improve enjoyment and PA in university students [46,49]. Both Interventions include theory-based motivational messages and information about PA, while one included sharing their activities and progress with the other participants via Facebook [49]. The other study gave out pedometers to one of the two intervention groups to track their process individually. Even though the intervention only lasted three weeks, the combination of messages and using a pedometer increased enjoyment (η² = 0.09, *p* = 0.044) as well as daily steps (η² = 0.22, *p* < 0.001) in comparison to the control condition [46]. Participants receiving the Facebook intervention were additionally allocated to either participate in a weekly physical fitness class or to take part in a voluntary exercise program with free access to training facilities. Whilst within the more autonomous group enjoyment improved (η² = 0.34, *p* < 0.01), only in the weekly fitness class group did PA increase (η² = 0.41, *p* < 0.001) [49].

#### 3.3.4. Audio- and Video-Based Exergaming Programs

In two of the three exergaming interventions, children played active video games in a pre-school [38] or in an after-school program [39]. The third study compared the use of an immersive running app (Zombies, Run, Six to Start, London, UK) in adolescents to a non-immersive running app and a control condition [50]. None of the studies are explicitly theory-based and sample sizes are small. All fail to improve enjoyment, and only one intervention improving PA (*d* = 0.68, *p* = 0.003) required children to play active video games for half an hour five times a week [38]. 

## 4. Discussion

The first purpose of this paper was to conduct a systematic review on interventions to promote positive affect and PA to children, adolescents and young adults. The hypothesis, that interventions influence enjoyment, was supported by significant results in 7 out of the 13 included studies [40,43,44,45,46,48,49]. Our second hypothesis, that interventions influence PA behavior, was supported by six studies [38,44,45,46,48,49], though not all studies show effects on both enjoyment and PA. One study conducted a mediator analysis which found a significant effect from the intervention on PA through enjoyment [43]. Therefore, most interventions included in this review were only partly successful in changing enjoyment and PA behavior. These different results may be due to different theoretical approaches or to the lack of theory, the variety of intervention components and their combination, how the study was set up and how the data was collected and analyzed.

### 4.1. Theoretical Foundation of Interventions 

While some studies in this review provided a theoretical link for each individual component [43], others were not theory-based nor stated solid empirical evidence and a comprehensive rationale on why and how positive affect is targeted in the intervention. Specifically, none of the video- and audio-based exergaming interventions were truly affect-based and failed to improve enjoyment [38,39,50]. Therefore, future studies should be based on theories that can link the interventions to a change in affect and PA behavior.

The most commonly used theory in this review is the SDT, and most studies incorporating it improve both PA and enjoyment [43,45,49]. The theory states that intrinsically motivated behaviors cause enjoyment [19] and that specifically, the satisfaction of the basic psychological needs leads to enjoyment. In the PA context, autonomy [21], competence [20,23] and relatedness [24] can predict enjoyment in children and adolescents. Therefore, the SDT can serve as a solid theoretical foundation for interventions, even though it does not focus directly on affect. 

In addition, new affect-based models and frameworks can be incorporated to strengthen the theoretical foundation. For example, emotional triggers in the model of emotional perception in PE, which are found to be similar to the basic psychological needs, can provide further starting points. Attractiveness of the task, which is strongly influenced by previous experiences, might be a fourth factor to focus on in PE, besides autonomy, perceived competence and social belonging [26]. 

Another new theoretical approach is the affective-reflective theory by Brand and Ekkekakis [17]. The theory postulates that momentary affect can influence behavioral decisions in a fast and automated manner, while for a long-term PA behavior change, a cognitive reflection is needed. Even though none of the included intervention studies were based on the affective-reflective theory, studies which integrate automated valuation and cognitive reflection on affect can bring new insights and can indicate if this theory is suitable for studies with children, adolescents and young adults. 

Several studies use multiple theories to base their intervention upon and so are able to appropriately address each component and their interaction. For example, Murillo Pardo et al. [45] use the SDT to explain behavior change on a psychological, intra-personal level and, additionally, the Social Ecological Model that explains how the interventions targeting personal, social and environmental level interact and create a change in enjoyment and PA behavior. Therefore, when planning interventions, several theories can and should be used to design effective multi-component concepts [51]. 

### 4.2. Intervention Components—Reviewing the Evidence

Even though most group-based programs did not explicitly state theories, enjoyment increased in established physical activity programs that focus on fun activities [40,44]. Two further studies could not increase enjoyment and only have a brief description of intervention content [41,42]. Detailed descriptions of interventions are needed to compare and discuss findings.

Task-oriented teaching styles were a part of effective studies in PE and more comprehensive studies [44,45,48], whereas only one study reported no effects on enjoyment and PA [47]. By creating a motivational climate where students focus on the mastery of different tasks instead of comparing themselves to others, autonomy, competence and relatedness can be influenced positively which leads to higher enjoyment [20,21,52,53] and increased PA [21]. Teachers and instructors should therefore be included in the study design of interventions that involve instructed PA to focus on task-oriented teaching styles.

Voluntary PA programs and opportunities for PA, e.g., during recess or after school, were provided in several studies which were effective in increasing enjoyment [43,45,49] and PA [45]. Such autonomy-supportive offers can enhance students’ enjoyment [54], as participating in many different physical activities is associated with higher enjoyment [55]. One study [45] let students create their own PA program to actively improve their sense of autonomy and to provide higher enjoyment. While intrinsic motivation leads to positive affective experiences [19], extrinsic motivation can inhibit the future intention to exercise [56]. Therefore, supporting participants’ autonomy should be a part of interventions to promote enjoyment and PA.

Several included studies applied text affective messages or instrumental information via internet platforms or social media to motivate participants and were successful in changing enjoyment and PA [43,45,46,49]. In comparison, affective messages seem to be able to influence PA behavior more strongly than messages with instrumental information [57]. Most studies implemented both affective and informational messages but did not explicitly state how they aimed to increase enjoyment through these messages. Therefore, how the messages and the way of delivery influence enjoyment remains unclear in these interventions. While technology-based interventions are effective in increasing PA in children and adolescents [58,59], only few assess a possible mediation of enjoyment and motivation [60,61]. Therefore, affect-based approaches in interventions using text messages or social media should be further investigated in more detail and in combination with other components. For example, one study additionally used pedometers to supplement motivational messages [46], which are found to be a good way to improve PA in children and adolescents [62], showing a way to combine different intervention components. 

Even though in this review exergames were not able to increase enjoyment [38,39,50], they are often associated with positive emotions as games are meant to be inherently fun [63]. Some exergames are enjoyed more than PA [64] and therefore, still post an attractive approach to promote PA. However, a theory-based and evidence-driven approach is needed to design effective exergaming interventions.

### 4.3. Methodical Limitations of Included Studies

Different study designs without randomization or control conditions and inappropriate sample sizes as well as the lack of a comprehensible description of the intervention content limit the quality of several studies. Some studies in this review have very small sample sizes (e.g., Reference [39]) and are therefore not able to find significant effects despite having an effective intervention. To prevent this in future studies, power analyses (e.g., References [44,50]) prior to the intervention help to develop an appropriate sample size for expected effects. Missing control groups (e.g., Reference [42]) limit the validity of the results as the effects cannot be unambiguously traced back to the treatment. Control groups and randomization on participant, class or school level improve the validity of the study. 

The most common reason for reduced study quality in this review was the lack of controlling confounders [38,39,40,41,42,43,44,45,46,47,49,50]. To improve internal validity of studies, confounders need to be addressed either in the study design or in the statistical analysis. Only one study in this review conducted a mediator analysis (showing an intervention effect on PA behavior through enjoyment) [43]. Mediator analyses are needed to trace back whether the intervention was effective in changing PA behavior due to a change in affect or other variables addressed in the intervention.

All studies used valid and reliable instruments to measure enjoyment and PA. For enjoyment, the Physical Activity Enjoyment Scale (PACES) and the enjoyment subscale of the Intrinsic Motivation Inventory (IMI) are the most commonly used questionnaires. Future studies should consider using these instruments to ensure a good comparability between studies. For PA, either objective or subjective measurements were used. While questionnaires are a cost-efficient way to measure PA behavior, objective measurements of PA are not influenced by recall bias or social desirability. Additionally, pedometers or accelerometers can be incorporated into interventions [46,62]. 

The duration of interventions ranges from three weeks to three years. While shorter interventions focus on few components only, larger studies tend to have more comprehensive approaches. The intervention length in this sample was not related to its outcome. Independently, follow-up measurements (e.g., References [40,43,46]) should be performed to examine long-term changes in enjoyment and PA behavior. 

### 4.4. Guidelines for Affect-Based Interventions

This review shows different approaches on how to promote positive affect and PA for children, adolescents and young adults. The second aim of this review is to provide guidelines for future affect-based studies in the light of previous research which are summarized here to improve the quality and validity of future studies. This allows for a better comparability of studies and enables the conduction of a meta-analysis.

- SDT is the most established theory, but new, more affect-oriented theories should be integrated into interventions to test and develop new approaches.
- Evidence points to effective components, especially task-oriented teaching styles and creating opportunities for voluntary PA. Still, high-quality studies are needed to further solidify the evidence. To what extent text messages, the use of social media, pedometers or exergames promote PA enjoyment should be investigated in further studies.
- Adequate sample sizes for randomized, controlled interventions with controlled confounders increase the validity of studies. Further, the use of established measurement methods and mediator analyses improve the study quality and build a foundation for convincing evidence to be summarized and reviewed in meta-analyses.

### 4.5. Limitations

The systematic search was conducted in two databases only, and for that reason, a thorough snowball search was carried out. This review identified 13 studies which led to a small number of studies for adolescents and young adults, making comparisons within these groups unfeasible. While this review can highlight different aspects of included studies, it was not deemed appropriate to conduct a meta-analysis due to a limited, heterogenous sample. As only one study conducted a mediator analysis, no causal relations between intervention, changes in enjoyment and PA can be shown for most studies. Several studies had a small number of participants which increases the susceptibility to a type II error, meaning that effective interventions might not have found significant effects. 

## 5. Conclusions

This review shows moderate evidence on interventions for children, adolescents and young adults to be effective in increasing enjoyment and PA. Over half of the interventions were not adequately based on affect-centered theories. The SDT was the most frequently used theory in our sample and while this theory is a good starting point to target enjoyment, more affect-based theories should be adapted in research to include other aspects of affective experiences. Especially, group-based PA programs incorporating task-oriented teaching styles and creating opportunities for voluntary PA were effective and can be recommended for practical application. The use of text messages, social media, pedometers or exergames to promote enjoyment and PA show inconclusive results in a small sample of studies. For most studies, it remains unclear how a change in enjoyment influences PA behavior due to missing mediator analyses. Further, a lack of control conditions and small sample sizes limit the validity of several studies. Future studies should focus on theory-based, multi-component interventions with mediator analyses and follow-up measurements to build strong evidence for sustainable affect-based interventions to promote PA.

## Figures and Tables

**Table 1:** Characteristics of included studies.

Characteristics	Samples N (%)
*Study design* Randomized controlled trials Controlled trials Cohort	5 (39) 4 (31) 4 (31)
*Gender* Only female Mixed	3 (23) 10 (77)
*Age groups* 4–13 years 14–17 years 18–23 years	8 (62) 2 (15) 3 (23)
*Intervention setting* School University Church community Not specified	8 (62) 3 (23) 1 (8) 1 (8)
*Theory* SDT Trans-Theoretical Model SDT + Health Promotion Model SDT + Social Ecological Model SDT + Theory of Planned Behavior Achievement Goal Theory + Social Ecological Model None	1 (8) 1 (8) 1 (8) 1 (8) 1 (8) 1 (8) 7 (54)
*Measurements of enjoyment (self-reported)* PACES/sPACES Enjoyment subscale of IMI PE enjoyment scale PA enjoyment scale Enjoyment subscale of MPAM-R PACES + Enjoyment subscale of IMI	6 (46) 3 (23) 1 (8) 1 (8) 1 (8) 1 (8)
*Measurements of PA* Accelerometer Pedometer Questionnaire Questionnaire + accelerometer	4 (31) 3 (23) 5 (39) 1 (8)

SDT: Self-Determination Theory; PACES: Physical Activity Enjoyment Scale; s-PACES: short Physical Activity Enjoyment Scale; IMI: Intrinsic Motivation Inventory; MPAM-R: Motivation for Physical Activity Measure—Revised.

**Table 2:** Data extraction.

Source (Author, Year, Country of Origin)	Study Design (Theory, Measurement Points, Statistics)	Sample (Setting, Sample Size, Mean Age)	Intervention (Length of Intervention, Treatment, Duration, Frequency)	Outcome (Measurements of Affect and PA)	Results
Direito et al., 2015 [50], New Zealand	RCT baseline, post-test (8 weeks) ANCOVA	adolescents n = 51 (57% female) M = 15.7 ± 1.2 years	8 weeks (1) immersive running app (n = 17), 3x/week (2) non-immersive running app (n = 16), 3x/week (3) control: sport as usual (n = 18)	enjoyment: PACES PA: PAQ-A, accelerometer (for following 7 days after each assessment point)	enjoyment (n.s.) PA (n.s.)
Fairclough et al., 2016 [44], Ireland	CT baseline, mid- (3 weeks), post-test (6 weeks) Oct. 2015–Dec. 2015 ANOVA, ANCOVA	4 co-educational primary schools n = 139 M = 10.7 ± 0.6 years	6 weeks (1) Born to Move (n = 73): class-based PA program with music, 8 movement categories for motor skills 2x/week (30 min) and regular PE lesson 1x/week (30–45 min) (2) control (n = 66) regular PE lessons 2x/week (30–45 minutes)	enjoyment: short-form IMI (baseline, mid-test) PA: LPA, MPA, MVPA, VPA, total PAsedentary time: accelerometers	group x time interaction for enjoyment in (1) (*p* = 0.049) increased enjoyment in (1) at mid-test (*d* = 0.56, *p* = 0.02) increased LPA (*d* =0.21, *p* = 0.006), MPA (*d* = 0.15, *p* = 0.026), MVPA (*d* = 0.14, *p* = 0.044), total PA (*d* = 0.18, *p* = 0.033) in (1) VPA (n.s.) decreased sedentary activity (*d* = 0.39, *p* = 0.008)
Fu et al., 2018 [38], USA	RCT post-test (12 weeks) MANOVA	preschool n = 64 (48% female) M = 4.9 ± 0.7 years	12 weeks (1) Exergames (n = 35) 15 min “GoNoodle”, 10 min “Adventure to Fitness”, 5 min “Cosmic Kids Yoga” (2) control (n = 29): active free play 5x/week (30 min)	enjoyment: subscale of IMI PA: pedometer	enjoyment (n.s.) PA higher in (1) at post-test than in (2) (*d* = 0.68, *p* = 0.003)
George et al., 2015 [39], Canada	Cohort Pre- and post-test (6 weeks) t-tests, regression analysis, rANOVA	after school program n = 15 (53% female) M = 7.9 ± 2.12	6 weeks (1) 4 active video games from the Nintendo Wii system: Wii Sport, Wii Sport Resort, Wii Play and Just Dance 2 (self-selected) 2x/week (20 min or more)	enjoyment: PACES, subscale of IMI PA: pedometer	enjoyment: PACES (n.s.), IMI (n.s.) PA (n.s.)
Grâsten and Yli-Piipari, 2019 [47], Finland	Achievement Goal Theory, Social Ecological Model CT t0 (spring 2012), t1 (spring 2013), t2 (spring 2014) latent growth curve models	Elementary school children n = 661 (1) M = 12.23 ± 0.42 (2) M =12.04 ± 0.21	2 years (1) Physical Activity as Civil Skill Program (n = 265): task-involving teaching practices in PE, developing physical environment and providing equipment for PA during breaks 90 min PE/week (2) control (n = 396): regular PE 90 min PE/week	enjoyment: PE Enjoyment Scale MVPA: HBSC	No change in PE enjoyment over time in (1) (n.s.) and (2) (n.s.) No time x group interaction effect (n.s.) No relationship between MVPA and enjoyment (n.s.)
Huberty et al. [40], 2014, USA	Cohort Baseline, post-test (12 weeks), follow up (26 weeks) Friedman test, ANOVA	7 after school programs n = 182 (100% female) (1) n = 33 (M = 6.4 ± 0.7) (2) n = 90 (M = 9.2 ± 0.8) (3) n = 59 (M = 11.3 ± 0.7)	12 weeks (1) GoGirlGo: storytelling and discussion (30 min) and PA (30 min) 1 h/week	enjoyment: s-PACES MVPA: accelerometry	increase in enjoyment from baseline to follow-up (*p* = 0.016), but not from baseline to post-test (n.s.) MVPA from baseline to follow-up n.s.
Invernizzi et al., 2019 [48], Italy	RCT baseline, post-test ANCOVA	fifth-grade students n = 121 (52% female) M = 10.5 ± 0.5	12 weeks (1) n = 62 PE program based on multi teaching approach by graduate PE students (2) n = 59 standard PE lessons by primary teachers 2 h per week	enjoyment: PACES PA: PAQ-C	difference of enjoyment between (1) and (2) (η² = 0.96, *p* < 0.001) higher increase of PA in (1) compared to (2) (η² = 0.09, *p* = 0.002)
Miragall et al., 2017 [46], Spain	TTM RCT baseline, post-test (3 weeks), follow-up (after 3 months) ANCOVAs (controlled for baseline)	inactive university students n = 76 (86% female) M = 22.18 ± 3.71	3 weeks (1) IMI (n = 24): internet-based motivational intervention (2) IMI + PED (n = 26): IMI and access to data of pedometer (3) control (n = 26)	enjoyment: s-PACES steps: pedometer	Group main effect in enjoyment (η² = 0.09, *p* = 0.044), with higher enjoyment in (2) than in (3) (*p* = 0.038) Group main effect in daily steps (η² = 0.22, *p* < 0.001), with more daily steps in (2) than (3) (*p* < 0.001)
Murillo Pardo et al., 2016 [45], Spain	SDT, Social Ecological Model CT baseline (Oct. 2009), t1 (Oct. 2010), t2 (Oct. 2011), t3 (May 2012) linear mixed models	4 secondary schoolsn = 553 (47% female) (1) n = 204, 2009–2012 (M = 12.03 ± 0.16) (2) n = 176, 2010–2012 (M = 12.07 ± 0.26) (3) n = 173, 2011–2012 (M = 12.05 ± 0.21)	1–3 academic years (1) Sigue la Huella: 2 schools (n = 302) multi-component strategy for PE promotion through tutorial action, school PE, information dissemination, participation in institutional and special programs (2) control: 2 schools (n = 251)	enjoyment: PACES MVPA: accelerometer (at least 4 days/week)	positive intervention effect on enjoyment in (1) (*β* = 0.11, *p* < 0.001) enjoyment of PA as predictor of MVPA approaches significance (*β* = 2.22, *p* = 0.080) time x group interaction effect in MVPA (*β* = 8.51, *p* < 0.001)
Quartiroli and Maeda, 2016 [41], USA	SDT, TPB Cohortbaseline, post-test paired t-tests	university students n = 58 (57% female) M = 18.72 ± 1.09	15 weeks (1) 4 sections of a lifetime physical fitness course 3x/week (50 min)	enjoyment: subscale of MPAM-R PA: MPA, VPA, SPA, STPA: sIPAQ	enjoyment (n.s.) PA: MPA (n.s.), VPA (n.s.), SPA (n.s.)
Robbins et al., 2019 [43], USA	SDT, HPM RCT baseline, post-test (17 weeks), follow-up (9 month) path analysis models	24 secondary schools n = 1519 (100% female) M = 12.05 ± 1.01	17 weeks (1) 12 schools (n = 753) Face-to-face motivational interviewing sessions (begin and end of intervention), after-school PA club offering fun PA opportunities with coaches 3x/week, individually tailored motivational and feedback messages at intervention midpoint (2) control: 12 schools (n = 766)	enjoyment: PA Enjoyment Scale PA: accelerometer (7 days)	positive intervention effect on enjoyment in (1) (*B* = 0.07, *p* = 0.047) changes in enjoyment had a positive effect on MVPA change from baseline to post-test (*B* = 24.48, *p* < 0.001) negative influence of enjoyment on MVPA change from post-test to follow-up (*B* = –13.83, *p* < 0.001)
Thompson et al., 2013 [42], USA	Cohort baseline, mid- (6 weeks), post-test (12 weeks)t-test, rANOVA	African American church community n = 39 (100% female) M = 14.2 ± 1.6	12 weeks (1) Fitness U N -joy project: group discussion focusing at attitude, self-efficacy, enjoyment (30 min) and interactive dance aerobic class (30 min) 1 day/week	enjoyment: PACES PA: APARQ	enjoyment (n.s.) decreasing PA over time (*p* = 0.01)
Wang et al., 2015 [49], Singapore	SDT CT baseline, post-test MANOVA	university students n = 62 (50% female) M = 22.30 ± 1.51	8 weeks (1) fitness class (n = 17): 1 h lecture, 1 h introduction to set of exercises, 1 h exercising by themselves 1 day/week (2) fitness class as in (1) (n = 14) + Facebook: motivational messages and information about PA, opportunities for interaction 1 day/week (3) exercise program + Facebook as in (2) (n = 24): 1h aerobic exercises with researcher (voluntarily), training beyond aerobic lesson in training room possible 1 day/week (4) control (n = 7)	enjoyment: subscale of IMI PA: IPAQ	time x group interaction effect in enjoyment (η² = 0.34, *p* < 0.01) with higher enjoyment in (3) than in other groups time x group interaction in PA (η² = 0.41, *p* < 0.001) with higher PA in (1) and (2) than in (3) and (4)

TTM: Trans-Theoretical Model; SDT: Self-Determination Theory; TPB: Theory of Planned Behavior; HPM: Health Promotion Model; RCT: randomized controlled trial; CT: controlled trial; ANOVA: Analysis of Variance; rANOVA: repeated measure ANOVA; ANCOVA: Analysis of Covariance; MANOVA: Multivariate Analysis of Variance; PA: physical activity; PE: physical education; LPA: light physical activity, MPA: moderate physical activity; MVPA: moderate-vigorous physical activity; VPA: vigorous physical activity; SPA: strength physical activity; STPA: stretching; PAQ-A: Physical Activity Questionnaire; sIPAQ: short International Physical Activity Questionnaire; APARQ: Adolescent Physical Activity Recall Questionnaire; PACES: Physical Activity Enjoyment Scale; s-PACES: short Physical Activity Enjoyment Scale; IMI: Intrinsic Motivation Inventory; MPAM-R: Motivation for Physical Activity Measure—Revised; HBSC: Health Behavior in School-aged Children Research Protocol; PAQ-C: Physical Activity Questionnaire for Older Children; n.s: not significant.
